# A Nap But Not Rest or Activity Consolidates Language Learning

**DOI:** 10.3389/fpsyg.2017.00665

**Published:** 2017-05-16

**Authors:** Stefan Heim, Juliane Klann, Kerstin I. Schattka, Sonja Bauhoff, Gesa Borcherding, Nicole Nosbüsch, Linda Struth, Ferdinand C. Binkofski, Cornelius J. Werner

**Affiliations:** ^1^Department of Psychiatry, Psychotherapy and Psychosomatics, Medical Faculty, RWTH Aachen UniversityAachen, Germany; ^2^Research Centre Jülich, Institute of Neuroscience and Medicine (INM-1)Jülich, Germany; ^3^Department of Neurology, Medical Faculty, RWTH Aachen UniversityAachen, Germany; ^4^SRH University of Applied Health Sciences GeraGera, Germany; ^5^Division for Clinical Cognitive Sciences, Department of Neurology, Medical Faculty, RWTH Aachen UniversityAachen, Germany; ^6^Research Centre Jülich, Institute of Neuroscience and Medicine (INM-4)Jülich, Germany

**Keywords:** sleep, rest, words, interference, aphasia, memory, consolidation

## Abstract

Recent evidence suggests that a period of sleep after a motor learning task is a relevant factor for memory consolidation. However, it is yet open whether this also holds true for language-related learning. Therefore, the present study compared the short- and long-term effects of a daytime nap, rest, or an activity task after vocabulary learning on learning outcome. Thirty healthy subjects were divided into three treatment groups. Each group received a pseudo-word learning task in which pictures of monsters were associated with unique pseudo-word names. At the end of the learning block a first test was administered. Then, one group went for a 90-min nap, one for a waking rest period, and one for a resting session with interfering activity at the end during which a new set of monster names was to be learned. After this block, all groups performed a first re-test of the names that they initially learned. On the morning of the following day, a second re-test was administered to all groups. The nap group showed significant improvement from test to re-test and a stable performance onto the second re-test. In contrast, the rest and the interference groups showed decline in performance from test to re-test, with persistently low performance at re-test 2. The 3 (GROUP) × 3 (TIME) ANOVA revealed a significant interaction, indicating that the type of activity (nap/rest/interfering action) after initial learning actually had an influence on the memory outcome. These data are discussed with respect to translation to clinical settings with suggestions for improvement of intervention outcome after speech-language therapy if it is followed by a nap rather than interfering activity.

## Introduction

“To sleep, perchance to dream; aye, there’s the rub.”

(William Shakespeare: Hamlet, Act III, Scene I).

Human adults spend about one third of their lifetime asleep (Alger et al., 2015). This state of reduced consciousness is a useful mechanism not only for physiological recreation but also for the consolidation of memory traces (Rechtschaffen and Kales, 1968; Diekelmann and Born, 2010; Diekelmann, 2014). During nocturnal periods of sleep^1^, novel words and concepts get integrated into the existing semantic networks (Wang et al., 2016). In fact, there is a direct relationship between the duration of sleep and the amount of learning (Earle et al., 2017). On the other hand, patients with sleep disorders often suffer from impaired memory (Cellini, 2016). Moreover, there seems to be a distinction between procedural (implicit; not verbalisable; unintentional; often tested with motor sequences) and declarative (explicit, verbally expressible; intentional; factual) types of learning and memory (e.g., Squire and Zola, 1996).^2^ Procedural learning (e.g., finger tapping sequences) relies more on phases of rapid eye movement (REM) sleep while declarative contents such as word association learning depends more on phases with sleep spindles (Philal and Born, 1997; for a recent review of the electrophysiological account of sleep-induced memory consolidation, which will not be part of the present paper, cf., e.g., Chatburn et al., 2014; or Rasch and Born, 2013). While both younger and aged subjects show such sleep-dependent memory consolidation for declarative contents, it is only the younger subjects for whom also procedural learning is supported (Pace-Schott and Spencer, 2015). The performance of healthy elderly participants in a procedural learning task after sleep is comparable to that after a waking period – only elderly stroke patients actually revealed positive effects of sleep after procedural motor learning (Backhaus et al., 2015; see also Gudberg and Johansen-Berg, 2015).

Importantly, it is not only nocturnal sleep that has a positive influence on memory consolidation. Short diurnal periods of sleep, i.e., naps, seem to exert positive influence on procedural/motor learning (e.g., Nishida and Walker, 2007; Seeck-Hirschner et al., 2010) and declarative/associative learning (e.g., Lahl et al., 2008). These supportive effects are particularly pronounced when the learning phase is followed immediately by the nap (Benson and Feinberg, 1977; Gais et al., 2006; de Bruin et al., 2016). When directly compared to a matched no-nap control group, a group enjoying a 70-min nap outperformed their controls at re-test in a procedural learning (juggling) experiment (Morita et al., 2016) and in a declarative (picture memory) study (Cellini, 2016).

However, other recent studies shed some doubt on the supportive role of a nap for motor learning. Although the meta-analysis by Pan and Rickard (2015) could confirm the overall effect of a bigger gain in groups with vs. without nap, the authors could explain that gain by the influence of moderator variables such as time of testing or training duration, rather than the sleep *per se*. In two studies using sequence learning and motor adaptation in a cross-over design, subjects were randomly assigned to wake, short nap, or long nap groups. The authors did not find any effect of sleep condition in either task.

Thus, it appears that for procedural motor learning, the debate cannot be concluded at this point. However, for declarative learning, in particular for language learning, the debate about the beneficial role of naps, i.e., shorter day-time sleeps with a maximum of one REM cycle, seems not to have even started yet. While it is widely agreed that (night) sleep positively impacts on vocabulary acquisition in infants (e.g., Axelsson et al., 2016), recent research in adults preferentially addresses the question of reduced amount and quality of overall sleep in older subjects on language learning (Kurdziel et al., 2016), or how learning during sleeping can be improved, e.g., by exposure of the sleepers to relevant foreign language cues (Schreiner and Rasch, 2016).

It is thus an open issue whether the relative benefit of a nap over waking, which is presently being disputed in the domain of procedural motor learning, can be found for vocabulary learning as a particular instance of declarative learning. Therefore, the present study addressed this question directly, comparing three groups of elderly healthy adults that completed a pseudo-word learning task followed by (a) nap, (b) passive rest, or (c) an interfering activity. By including elderly instead of younger adults, the study taps in particular into the distinction of procedural vs. declarative learning, since, as discussed above, older subjects’ procedural learning is potentially unaffected by sleep or nap, whereas an influence on declarative language learning can be hypothesized (cf. Backhaus et al., 2015; Gudberg and Johansen-Berg, 2015; Cellini, 2016). If sleep in fact has a positive impact on the consolidation of language learning, this mechanism would be of potential use for the rehabilitation of aphasia after stroke, a condition that affects older people much more than young.

## Materials and Methods

All procedures were approved by the Institutional Review Board of the Medical Faculty of RWTH Aachen University.

### Participants

For the study, healthy elderly subjects between 50 and 75 years of age were recruited. They grew up monolingually, with German as their native language and at least a high school degree (9 years of school; German “Hauptschulabschluss”). All subjects had normal or corrected-to-normal audition and vision. Exclusion criteria were a known history of psychiatric and/or neurodegenerative disease or other cognitive disability, regular intake of medication with potential effect on quality or quantity of sleep, and abuse of drugs or alcohol. Further exclusion criteria were acute sleep disorders, work in shifts, or participation in a foreign language class up to 6 months prior to the experiment.

A total of 30 volunteers were included in the study, forming three groups of *n* = 10 subjects each. The first group was going to have a nap, the second a phase of active rest, and the third an interfering activity task.

All groups were comparable with respect to years of education, age, level of day sleepiness, and daily amount of caffeine intake (**Table 1**). The number of women was equally distributed among the groups [Freeman-Halton extension of the Fisher exact probability test for a 2-rows by 3-columns (instead of the typical 2 × 2) contingency table].^3^

**Table 1:** Characterization of the three experimental groups (mean ± SEM).

Variable [unit]	Nap	Rest	Interference	*p*
Age (years)	62.6 (1.8)	59.9 (1.6)	60.0 (1.5)	0.432
Education (years0	11.9 (0.6)	10.4 (0.6)	11.7 (0.5)	0.148
Day sleepiness^∗^ (ESS score)	4.4 (0.8)	6.5 (0.9)	4.7 (0.8)	0.181
Daily caffeine intake (cups)	4.2 (0.8)	4.4 (0.7)	2.6 (0.4)	0.136
*n* (female)	6	7	5	0.526
*n* (total)	10	10	10	

*The last column refers to the test of comparability between groups: one-way ANOVA for Age, Education, Sleepiness, and Caffeine; Freeman-Halton extension of the Fisher exact probability test for number of women per group. ^∗^ Epworth Sleepiness Scale (Johns, 1991).*

### Experimental Task

The core of the study was a pseudo-word learning task, after which the sample was split into three different interventions: daytime nap, active rest, or interference (see below). This pseudo-word learning task was designed as follows. A set of 24 images of fantasy “monsters” was obtained from www.shutterstock.com. Each monster was given a name that was a 1- to 3-syllabic pseudo-word in German, obtained by the exchange of one or more vowels from real German words (words taken from the dissertation by Mouson, 2009). An equal number of 1-, 2-, and 3-syllabic names was given to the 24 monsters.

**FIGURE 1:**
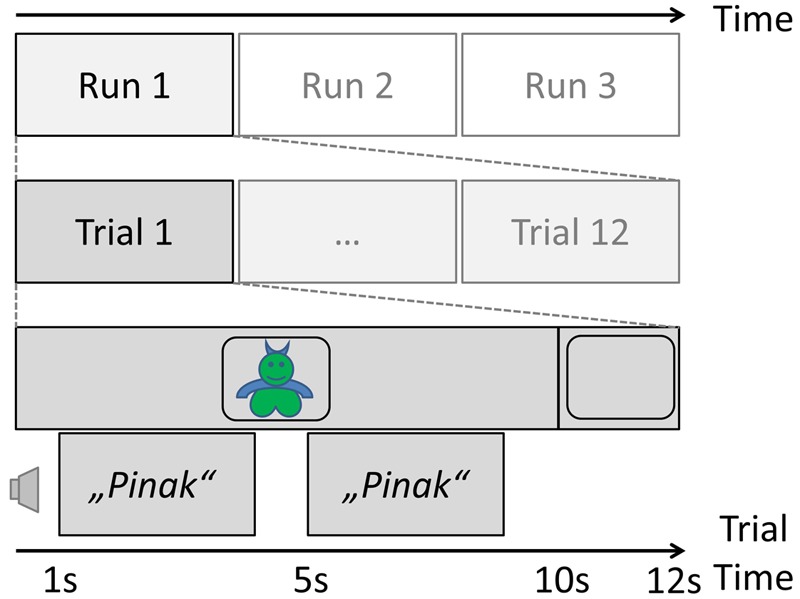
**Trial schema of the learning phase of the experiment before the nap/interference manipulation**.

This set was then divided into two sub-sets (A and B). The items of set A were used for the pseudo-word learning task prior to the intervention phase for all subjects. The experiment consisted of three runs, which were prepared by the in-house *Audio-Visual Media Center* as time-locked video (mpg) presentations for a laptop computer screen. During each run, all monsters were presented in a quiet, normally lit room in randomized order for 10 s each, with a 2-s inter-stimulus interval with a blank black screen. While the image of a monster was on the screen, its name was presented twice via loudspeakers connected to the laptop, once after 1 s and then again at second 5. A schematic of the learning phase of the study can be found in **Figure 1**. Subjects were instructed to memorize the name of the monster at that time.

### Study Design

Prior to participation, the subjects were informed about the study design and purpose, and the absence of any exclusion criteria was confirmed. All subjects signed the informed consent sheet. The experimental procedures were approved by the Institutional Revision Board of the Medical Faculty at RWTH Aachen University.

On Day 1, subjects were not allowed any caffeine or nicotine 2 h prior to the experiment. At 9:00 a.m., they were received by the experimenter and completed the sleepiness questionnaire. Then, at 9:30 a.m., the learning task was performed. At 9:45 a.m. their learning achievement was tested. The tests consisted of the presentation of each of images of the monsters in a pseudo-randomized order which differed at every test. Subjects were asked to recollect as many names as they could. They were not put under time pressure for their overt uncued naming response. The response was written down by the experimenter in a standardized protocol sheet and analyzed later for correctness by trained speech-language therapists. The data were cross-validated by at least one other rater.

Next, the subjects of the Nap group were offered the opportunity of a 90-min^4^ nap in a quiet room. The experimenter ensured that the subjects fell asleep and, if necessary, woke them up after 90 min. A polysomnographic examination was not conducted. The subjects of the Rest group were taken to a quiet room where they remained awake for 90 min painting mandalas, building Jenga^®^ towers or playing the Solitaire card game, i.e., non-verbal activities. They were not allowed any caffeine or nicotine during that time; only herbal infusions or mineral water were allowed. The Interference group received the same treatment as the Rest group with one modification: At the end of the rest period there was another pseudo-word learning task with a different set of stimuli for 10 min providing retroactive interference to the previous learning experience (cf. Korman et al., 2007). Then, all subjects performed the pseudo-word test a second time (*Re-Test 1*; 90-min re-test).

**FIGURE 2:**
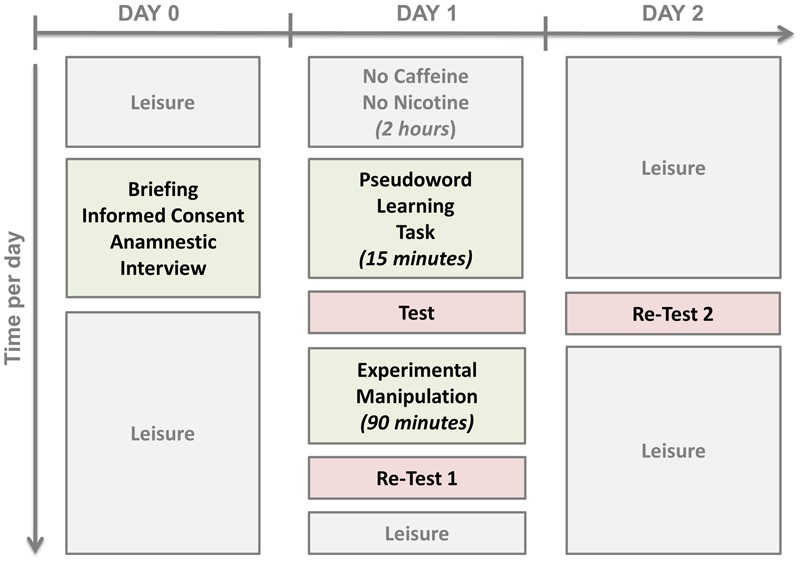
**Study Design**.

After that, all subjects went home. They came back on Day 2 at the same time as on Day 1 in order to perform the pseudo-word test again (*Re-Test 2*, 24-h re-test) in order to account for the effect of nocturnal sleep on the consolidation process. The study design is depicted in **Figure 2**.

### Statistical Analysis

Statistical analysis was performed with SPSS 20.0 (SPSS Corp., 2011). First, the initial level of performance of the three groups was compared to ensure equality of groups in this respect. Next, a 3 × 3 ANOVA with factors INTERVENTION (Nap/Rest/Interference) and TIME (Test/Re-Test1/Re-Test2) was conducted. Subsequently, planned pair-wise comparisons between Test, Re-Test1, and Re-Test2 were calculated individually for each group. The original (uncorrected) *p*-values as provided by SPSS are reported, but effects were only considered significant if they also survived Bonferroni correction. Finally, in order to understand the role of potential influence factors on the learning success, bivariate correlation coefficients were calculated in an exploratory manner.

## Results

**FIGURE 3:**
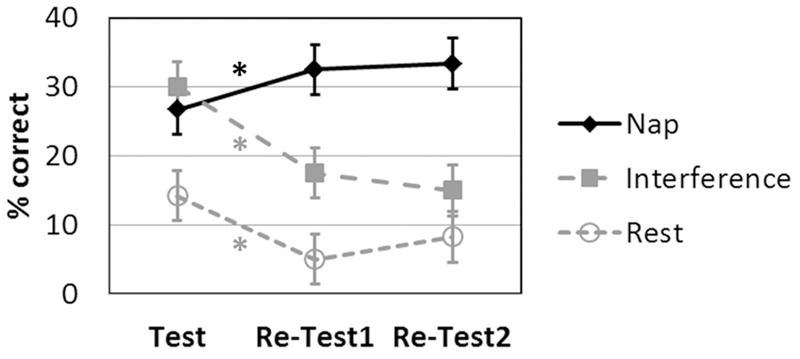
**Language learning achievement (recall) over time (Test: after the initial learning session; Re-Test1: immediately after nap/rest/interference; Re-Test2 on the next day) as a function of intervention.** ^∗^*p* < 0.05 Bonferroni-corrected. Symbols represent average percent correct performance per group, error bars indicate the standard errors of mean.

The language learning scores for the three groups at each time point are displayed in **Figure 3**.

### Comparison of Intervention Effects between Groups

The 3 × 3 ANOVA revealed significant main effects of INTERVENTION [*F*(2,27) = 10.147; *p =* 0.001] and TIME [*F*(2,54) = 10.473; *p <* 0.001] and a significant INTERVENTION × TIME interaction [*F*(4,54) = 15.374; *p* < 0.001].

### Planned Contrasts for the Nap Condition

The *post hoc* two-tailed dependent-sample *t*-tests for the Nap group revealed a significant increase in language learning from Test to Re-Test1 [*t*(9) = -3.280; *p* = 0.010] and from Test to Re-Test2 [*t*(9) = -3.207; *p* = 0.011]. There was no difference between Re-Test1 and Re-Test2 [*t*(9) = -0.557; *p* = 0.591 two-tailed].

### Planned Contrasts for the Rest Condition

The *post hoc* two-tailed dependent-sample *t*-tests for the Rest group revealed a significant decrease in language learning from Test to Re-Test1 [*t*(9) = 3.973; *p* = 0.003 two-tailed]. All other effects were not significant at a corrected level [Re-Test1 to Re-Test2: *t*(9) = -2.449; *p* = 0.037 two-tailed; Test to Re-Test2: *t*(9) = 2.090; *p* = 0.066 two-tailed].

### Planned Contrasts for the Interference Condition

The *post hoc* two-tailed dependent-sample *t*-tests for the Interference group revealed a significant decrease in language learning from Test to Re-Test1 [*t*(9) = 5.582; *p* < 0.001] and from Test to Re-Test2 [*t*(9) = 5.014; *p* = 0.001]. There was no difference between Re-Test1 and Re-Test2 [*t*(9) = 1.152; *p* = 0.279 two-tailed].

### Comparability of Groups before Intervention

In order to test whether the numerical differences in the initial level of performance after the pseudo-word learning session at the first Test had any influence on the results, a one-way ANOVA with factor INTERVENTION was conducted. This ANOVA showed a significant main effect [*F*(2,27) = 5.407; *p* = 0.011]. Subsequent *post hoc* comparisons (least square difference method, uncorrected for multiple comparisons providing maximum sensitivity for the existence of a difference under the assumption that no difference exists) revealed that the Rest group had lower performance than both other groups (Interference vs. Nap: *p* = 0.517; Interference vs. Rest: *p* = 0.004; Nap vs. Rest: *p* = 0.020).

### Comparison of Intervention Effects between Groups with Initial Performance as Covariate

Consequently, the original 3 × 3 ANOVA was re-run as an ANCOVA with the subjects’ individual performances at the first test as covariate. In this ANCOVA, the main effect of INTERVENTION was significant [*F*(2,26) = 23.000; *p <* 0.001]. Likewise, the INTERVENTION × TIME interaction remained significant [*F*(4,52) = 16.029; *p* < 0.001]. Only the main effect of TIME disappeared [*F*(2,52) = 0.241; *p =* 0.787].

### Correlation Analysis

The potential influence factors for learning outcome at Test (index T0), Re-Test1 (index T1), and Re-Test2 (index T2) were submitted into a series of correlation analyses with the initial test performance to identify any circumstances that might have differentially affected the later performance in the experiment. These factors were “years of education,” “biological age,” “day sleepiness,” and “caffeine intake.” The analysis revealed no systematic effects whatsoever: years of education (*p*_T0_ = 0.677; *p*_T1_ = 0.441; *p*_T2_ = 0.592), age (*p*_T0_ = 0.750; *p*_T1_ = 0.606; *p*_T2_ = 0.732), day sleepiness (*p*_T0_ = 0.228; *p*_T1_ = 0.272; *p*_T2_ = 0.631), caffeine intake (*p*_T0_ = 0.944; *p*_T1_ = 0.305; *p*_T2_ = 0.249).

## Discussion

The present study sought to investigate whether a day-time nap may have a beneficial effect on language (vocabulary) learning in elderly subjects as compared to 90 min of active rest with or without interfering activity. The motivation was twofold: For one, the theoretical issue emerged from the literature review which revealed a vivid debate in the realm of procedural/motor learning but no comparable wealth of data for declarative/language learning. Moreover, the issue is of clinical relevance for the design of rehabilitation of patients with aphasia after stroke: Should periods of sleep be introduced as parts of the therapy, rather than providing interference to the speech-language therapy by some other therapeutic activity (cf. Siengsukon and Boyd, 2008, for the relevance to include sleep phases in the therapeutic schedule)?

The findings are straight-forward. There is a clear interaction of intervention and time on the pseudo-word learning task performance. Subjects in the Nap group profited significantly in their performance, whereas the subjects in the Rest and the Interference groups showed a significant decline in performance instead that was not compensated by the subsequent night sleep. The data thus replicate and extend earlier work on the role of sleep and nap for declarative learning (word recall) by Lahl et al. (2008), demonstrating that the association of pictures with novel pseudo-word names can also benefit from a day-time nap. The present study thus opens a novel perspective for the clinical application where confrontation naming in aphasic patients with word finding difficulties is still a standard procedure. Taking into account the suggestion by Gudberg and Johansen-Berg (2015) for inclusion of sleep into the therapeutic schedule and the meta-analytic findings by Backhaus et al. (2015) that stroke patients may show sleep-supported learning, the data from the present study may initiate further research in the realm of speech-language therapy.

Another facet that this study adds to the existing literature is the juxtaposition of passive rest and active interference. Even though the data for these two non-nap groups show comparable temporal trajectories, it might be worth investigating further whether, and if so, at what point, interference might disturb consolidation more than mere rest (cf. Shadmehr and Brashers-Krug, 1997; Korman et al., 2007; for the protective effect of sleep *after* interference see Ellenbogen et al., 2007; or Ertelt et al., 2012). In the present study, the time interval between the learning task and Re-Test 1 was 10 min longer for the Interference group than for the Rest group due to the additional interference task and since the actual rest period was supposed to be comparable between groups, ensuring comparability also with the 90 min nap in the Nap group. Subsequent studies investigating the manipulation of the retention intervals between Test and Re-Test1 might provide additional insights into the stability of the effects.

Moreover, the present study also contributes to the distinction between procedural/motor and declarative/language learning, showing that several study designs from the former domain might be re-run in the latter. This holds in particular for the question whether older subjects can, or cannot, profit from the potentially consolidating effect of sleep on learning performance. The meta-analysis by Backhaus et al. (2015) and their subsequent empirical studies (Backhaus et al., 2016a,b) suggested that sleep has no such effect on older subjects in motor learning paradigms. The data presented here could be taken to indicate that the consolidation mechanisms behind declarative/language learning may be different to some extent (see, e.g., Philal and Born, 1997, or Gais and Born, 2004, for a discussion of the distinctive relevance of REM vs. slow-wave sleep for the consolidation in procedural vs. declarative learning tasks). Electrophysiological or neuroimaging models were not part of this study (cf. Chatburn et al., 2014; Peigneux, 2015; or Rothschild et al., 2016); however, the present study might serve as an inspiration to compare the neurophysiological pathways by which sleep differentially modulates procedural and declarative learning, and to what extent that knowledge may be translated to clinical application.

Despite the clear pattern of results, several potential limitations should be considered. For one, the sample size with *n* = 30 is not large. Even though all effects of the experimental manipulation and their interaction were significant, a higher power might help better distinguish consolidating effects also from the nocturnal sleep periods.^5^ This is of particular importance since other studies of procedural (Morita et al., 2016) and declarative (Cellini, 2016) learning also observed performance improvement in non-sleep control groups. One potential explanation for the absence of such positive learning effect in the present study could be derived from King et al. (2015; see discussion in King et al., 2017), who argue that a low performance level at the first test provides only little chance of consolidation.^6^ This was exactly the situation for the Rest group, whose initial performance was below that of the other two sub-groups. Even though we considered the initial performance level as a covariate, and despite the lack of any significant correlations of the demographic variables with test performance, it could be that higher performers would have better consolidation. Finally, subsequent studies might make use of cross-over designs for Nap/Rest/Interference to control for between-group differences not only statistically (as done here) but also by virtue of the study design itself.

## Conclusion

We were able to demonstrate that a day-time nap has a positive, consolidating effect on language (i.e., vocabulary or pseudo-word name) learning which exceeds effects of the same intensity of active rest or interfering activity, possibly due to the slow-wave sleep and/or REM sleep phases that are absent during mere rest. The clinical potential of this approach for speech-language therapy remains to be investigated both for the direct application but also for the theoretical background, e.g., in order to test how complex words or even syntactic utterances profit from naps, or how the degree of impairment of the patients (i.e., their pre-treatment level of performance interacts with the protective effects of sleep). For all these future directions, the present study provides a first stepping stone. Finally, it should be noted the present findings do not rule out (and were not indented to do so) the influence of other relevant factors on learning in the procedural or declarative domain or even their interaction, and their potential implications for the treatment of patients (cf., e.g., Schack et al., 2014, for a recent example of how motor imagery not only increases the efficacy of motor learning in healthy adults but may serve as a substitute for actual physical practice in injured participants).

## Ethics Statement

This study was carried out in accordance with the recommendations of Ethik-Kommission an der Medizinischen Fakultät der RWTH Aachen with written informed consent from all subjects. All subjects gave written informed consent in accordance with the Declaration of Helsinki. The protocol was approved by the Ethik-Kommission an der Medizinischen Fakultät der RWTH Aachen.

## Author Contributions

SH: Concept, study design, translation to clinical setting, data analysis, discussion, and writing of manuscript. JK: Concept, study design, data analysis, discussion, and revision of manuscript. KS: Concept, study design, concept for translation to clinical setting, data analysis, discussion, and revision of manuscript. SB: Study design, data acquisition, data analysis, discussion, and revision of manuscript. GB: Study design, data acquisition, data analysis, discussion, and revision of manuscript. NN: Study design, data acquisition, data analysis, discussion, and revision of manuscript. LS: Study design, concept for translation to clinical setting, discussion, and revision of manuscript. FB: Concept, study design, data analysis, discussion, and revision of manuscript. CW: Concept, study design, concept for translation to clinical setting, data analysis, discussion, and revision of manuscript.

## Conflict of Interest Statement

The authors declare that the research was conducted in the absence of any commercial or financial relationships that could be construed as a potential conflict of interest.

## Footnotes

1 Nocturnal sleep can usually be distinguished into different phases or cycles of 90–120 min, which can be further subdivided into Phases 1–4 representing increasing sleep depth, and a subsequent REM phase. The EEG reveals waves with frequencies <1Hz during slow-wave sleep, but theta rhythm (about 6–10 Hz) during REM phases. There are about 3–6 such cycles per night. There is still a lively debate about the neurophysiological mechanisms of learning and sleep (“sleep-assisted” vs. “Active System Consolidation” approaches) and their endocrinological basis (for a review cf. Gais and Born, 2004).

2 Note that there has been a debate for over 50 years about the definitions and distinctions of “procedural vs. declarative” vs. “implicit vs. explicit” learning and memory (e.g., Frensch and Runger, 2003; Kihlstrom et al., 2007), which is still on-going.

3 http://www.vassarstats.net

4 It appears that REM sleep deprivation affects learning more than slow-wave sleep deprivation (for a review see Curcio et al., 2006). Since one sleep cycle which ends with the REM sleep phase usually lasts 90 min, we decided to use this duration rather than that of 70 min (Cellini, 2016; Morita et al., 2016), during which time the relevant REM period might still be missing – even though the sleep cycles become shorter with increasing age (Schulz, 1997).

5 This is of particular importance for the subjects in the Rest group for whom a performance increase after night sleep was present and only failed to reach significance at a corrected level.

6 As to the overall comparably low performance at Test1, one has to keep in mind that arbitrary colored monsters were assigned meaningless, i.e., pseudo-word, names. The subjects did not learn “real” vocabulary of a novel language which could be integrated into an existing semantic network.
